# Upregulation of FEN1 Is Associated with the Tumor Progression and Prognosis of Hepatocellular Carcinoma

**DOI:** 10.1155/2020/2514090

**Published:** 2020-01-13

**Authors:** Yingying Zhang, Xin Liu, Liwen Liu, Jianan Chen, Qiuyue Hu, Shen Shen, Yongjian Zhou, Siyu Chen, Chen Xue, Guangying Cui, Zujiang Yu

**Affiliations:** ^1^Precision Medicine Center, The First Affiliated Hospital of Zhengzhou University, Zhengzhou 450052, China; ^2^Key Laboratory of Clinical Medicine, The First Affiliated Hospital of Zhengzhou University, Zhengzhou 450052, China

## Abstract

**Background:**

Studies show that patients with hepatocellular carcinoma (HCC) have poor prognosis, particularly when patients are diagnosed at late stages of the disease development. The flap endonuclease 1 (FEN1) gene is overexpressed in multiple malignant tumors and may promote tumor aggressiveness. However, its expression profile and functional roles in HCC are still unclear. Here, we evaluated the molecular mechanisms of FEN1 in HCC.

**Methods:**

The expression of FEN1 in HCC was evaluated using HCC mRNA expression data from TCGA and GEO databases. The expression of FEN1 was also confirmed by immunohistochemistry (IHC) using a tissue microarray (TMA) cohort with a total of 396 HCC patients. Kaplan-Meier analysis and univariate and multivariate Cox regression analyses were used to determine the correlation between FEN1 expression and survival rate of HCC patients. The molecular mechanism and biological functions of FEN1 in HCC were predicted using functional and pathway enrichment analysis *in vitro* experiments.

**Results:**

FEN1 was overexpressed in multiple HCC cohorts at both mRNA and protein levels. The receiver operating characteristic (ROC) curve showed that FEN1 can serve as a diagnostic predictor of HCC. Meanwhile, patients with high FEN1 expression levels showed lower overall survival (OS) and relapse-free survival (RFS) rates than those with low FEN1 expression. More importantly, we found that FEN1 elevation was an independent prognostic factor for OS and RFS in HCC patients based on univariate and multivariate analyses, indicating that FEN1 might be a potential prognostic marker in HCC. Furthermore, knocking down FEN1 resulted in suppressed cell proliferation and migration *in vitro*. This could have been due to regulation expressions of c-Myc, survivin, and cyclin D1 genes, indicating that FEN1 may function as an oncogene through its role in the cell cycle and DNA replication pathway.

**Conclusion:**

Our study indicated that high FEN1 expression might function as a biomarker for diagnosis and prognosis. In addition, the study confirms that FEN1 is an oncogene in HCC progression.

## 1. Introduction

Deaths resulting from hepatocellular carcinoma (HCC) have risen in the past decade necessitating numerous research activities across the world focusing on this condition [1]. Among the currently available treatments, surgery and liver transplantation are the mainstay treatments for early-stage HCC [2]. Unfortunately, HCC patients are often diagnosed during advanced stages of disease development rendering them ineligible for surgery because of insidious onset and early metastasis [3]. To counter this problem and boost chances of survival in these patients, biomarkers for early diagnosis and prediction of recurrence of the disease are urgently needed. Many lines of evidence have demonstrated that DNA damage is a causal factor for carcinogenesis and tumor progression. Particularly, inappropriate DNA repair can cause malignant transformation of cells by activating oncogenes or inactivating tumor suppressors [4]. Flap endonuclease 1 (FEN1), a structure-specific multifunctional enzyme, plays a pivotal role in both DNA replication and repair [5]. Previous studies have demonstrated that FEN1 single-nucleotide polymorphisms (SNPs) are involved in various cancer developments, including breast cancer [6], lung cancer [4, 7], hepatocellular carcinoma [8], esophageal cancer [9], and gastric cancer [10] as well as glioma [11]. However, expression profiles and functional analysis of the FEN1 gene have not been evaluated in HCC.

In the present study, we first determined the expression of FEN1 at both mRNA and protein levels on a relatively large scale. We then correlated the expression profiles with clinicopathological parameters and analyzed prognosis. The results showed that FEN1 expression in HCC tissues was highly elevated compared with its expression in healthy tissues. In addition, survival analysis revealed that high FEN1 expression was correlated with a low overall survival rate. Notably, bioinformatics analysis indicated that FEN1 might function as an oncogene by regulating the cell cycle and DNA replication pathway in HCC. Meanwhile, FEN1 knockdown inhibited cell proliferation and migration *in vitro.* These results indicated that FEN1 is a powerful and effective diagnostic and prognostic biomarker for HCC.

## 2. Materials and Methods

### 2.1. HCC Datasets

Sixteen HCC mRNA expression datasets (GSE6474, GSE10143, GSE39791, GSE45436, GSE14520, GSE36376, GSE54236, GSE60502, GSE76297, GSE76427, GSE62232, GSE64041, GSE77314, GSE84005, GSE84598, and GSE102083) were obtained from the Gene Expression Omnibus (GEO) database (http://www.ncbi.nlm.nih.gov/geo/). TCGA-LIHC and corresponding clinical data used in this study were downloaded from The Cancer Genome Atlas (TCGA) portal (https://gdc-portal.nci.nih.gov/).

### 2.2. Patients and Specimens

HCC tissue microarray (TMA) consists of 396 matched HCC samples, in which 341 include accessible follow-up data. Pan-cancer TMA contained lung cancer, renal cancer, esophageal cancer, thyroid cancer, stomach cancer, rectal cancer, breast cancer, cervical cancer, liver cancer, and colon cancer with a total of 400 cases. Approximately 20 pairs of each type of cancer tissues were used in the study. All these samples were acquired from the First Affiliated Hospital of Zhengzhou University, between April and December 2016 (ZZU TMA cohort). The study was permitted by the Ethical Committee of the First Affiliated Hospital of Zhengzhou University, and we recorded informed consent for all patients from whom data was collected.

### 2.3. Cell Lines and Culture

Human hepatocellular carcinoma cell lines (Hep-3b and Hep-G2) were bought from the Chinese Academy of Sciences (Shanghai, China). These cells were cultured in Dulbecco's modified Eagle's medium (DMEM) mixed with 10% fetal bovine serum (FBS) (Gibco, NY, USA) and 100 UI/ml penicillin/streptomycin (Gibco, NY, USA) in an incubator at room temperature, with 5% CO_2_ and 95% air. The cell lines used in the study were cultured for less than six months.

### 2.4. Oligonucleotides and Transfection

Lipofectamine 3000 (Invitrogen, CA, USA) was used to transfect FEN1-specific si-RNA and si-NC (GenePharma, Shanghai, China) into the HCC cells (in 6-well plates). At 48-72 hours posttransfection, cells were harvested and the transfection efficiency was determined by western blot (WB) analysis.

### 2.5. Cell Growth Assay

Cells (5000 per well) were plated into 96-well plates. Cell numbers were assessed using Cell Counting Kit-8 (CCK-8) (Dojindo, Japan) after 5 days of culture. Briefly, 10 microliters of the CCK-8 reagent was added into the cells and incubated at 37°C for 2 hours. Optical density of the mixture was determined at 450 nm using a spectrophotometer (Molecular Devices, CA, USA) in each well. The rate of DNA synthesis was evaluated with a 5-ethynyl-20-deoxyuridine (EdU) assay kit (RiboBio, Guangzhou, China). Images were taken and analyzed using a microscope (Tokyo, Japan) at ×40 magnification. Proliferative activity of cells depends on the proportion of EdU-stained (with red fluorescence) and Hoechst-stained (with blue fluorescence) cells. Approximately 1500 cells per well were seeded on 6-well plates; then, after 10 days of incubation, they were fixed with 4% paraformaldehyde for 30 minutes at room temperature followed by 1% crystal violet when colonies could be detected with a naked eye.

### 2.6. Cell Migration Assay

The rate of HCC cell migration was assessed using the wound healing test. Briefly, cells were first seeded into triplicate wells and grown to 40% confluence. Artificial gaps were then created using a 20 *μ*l pipette tip, and wounds and migration rates recorded at 0 and 48 h after scratching under an Olympus camera.

### 2.7. Western Blot Analysis

Protein was extracted using a radioimmunoprecipitation assay (RIPA) reagent (Beyotime) supplemented with a protease inhibitor cocktail (Roche, IN, USA). Protein concentration was measured using a BCA protein assay kit (Solarbio, Beijing, China); then, 30 *μ*g of the proteins was electrophoresed on 12% SDS-PAGE. The separated proteins were transferred onto polyvinylidene difluoride (PVDF) membranes (Millipore, USA) followed by the addition of primary antibodies. The reaction was blocked with 5% skim milk powder for one hour and then incubated overnight at 4°C. The membrane was washed and treated with corresponding secondary antibodies for one hour at room temperature. Finally, the photographic film was applied to expose the membrane. GAPDH was used as the internal control. The antibodies used in this study were the FEN1 antibody (1 : 1000, rabbit polyclonal, Proteintech Group, China) and GAPDH antibody (1 : 5000, mouse monoclonal Proteintech Group, China).

### 2.8. Immunohistochemistry (IHC) Analysis

Sample sections were briefly dewaxed and then rehydrated. A steam treatment was then done to retrieve the antigen before the sections were blocked with 3% hydrogen peroxide at 37°C for an hour. The contents were incubated with the primary antibody FEN1 (dilution 1 : 100, Proteintech Group, China) at 4°C overnight and biotinylated with a goat anti-rabbit secondary antibody (dilution 1 : 1000, Proteintech Group, China) for 30 minutes. Detection was done by SignalStain® DAB (CST, USA) while counterstaining was carried out using hematoxylin. Two pathologists independently evaluated the IHC scores in a blinded manner. Samples were scored based on the proportion of positive cells as follows: 1—none, 2—<25%, 3—25-50%, 4—50-75%, and 5—75-100%. Staining intensity was evaluated as follows: 0—none, 1—weak, 2—medium, and 3—strong. The total score was then calculated by multiplying the two subscores and the samples with total scores of 0-3 (1+), 4-6 (2+), 7-9 (3+), 10-12 (4+), and 13-15 (5+). Scores of 3+, 4+, and 5+ were regarded as having high FEN1 expression while those with 1+ and 2+ were deemed as having low expression.

### 2.9. Function Analysis

GSEA (gene set enrichment analysis) was performed to resolve the gene sets associated with FEN1 expression in TCGA dataset. Expression profiles of 328 samples from TCGA HCC dataset were divided into two groups based on expression levels of FEN1. The GSEA v2.0 software was used to reveal distribution of the gene sets in the MSigDB database v4.0.

### 2.10. Statistical Analyses

Statistical software, SPSS 23.0 software (IBM Corp., Armonk, NY, USA) and GraphPad Prism 7 (San Diego, CA, USA), was used for analysis. Correlation of FEN1 expression with clinical variables in HCC patients was estimated by the *χ*^2^ test or Fisher's exact test. Kaplan-Meier and log-rank tests were conducted for survival analysis. Student's *t*-test was used for comparison between groups with *P* values (two-sided) less than 0.05 considered statistically significant. Data were presented as means ± SD. All experiments were replicated three times.

## 3. Results

### 3.1. Expression Patterns of FEN1 in Various Human Cancers

**Figure 1:**
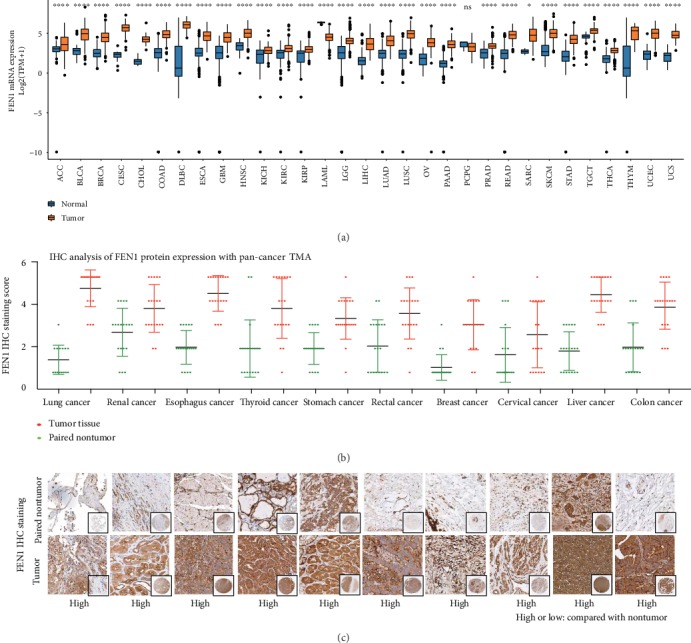
FEN1 expression is frequently upregulated in cancers. (a) FEN1 mRNA expression in TCGA data analysis in cancers and paired normal tissues. (b) FEN1 protein expression in pan-cancer tissues and paired nontumor samples. FEN1 protein was upregulated in tumor tissues. (c) Representative FEN1 histologic scoring in pan-cancer tissues and paired nontumor samples. FEN1: flap endonuclease 1; HCC: hepatocellular carcinoma; TMA: tissue microarrays; GEO: Gene Expression Omnibus; TCGA: The Cancer Genome Atlas; GSEA: gene set enrichment analysis; ACC: adrenocortical carcinoma; BLCA: bladder urothelial carcinoma; BRCA: breast cancer; CESC: cervical squamous cell carcinoma and endocervical adenocarcinoma; CHOL: cholangiocarcinoma; COAD: colon adenocarcinoma; DLBC: lymphoid neoplasm diffuse large B-cell lymphoma; ESCA: esophageal carcinoma; GBM: glioblastoma multiforme; HNSC: head and neck squamous cell carcinoma; KICH: kidney chromophobe; KIRC: kidney renal clear cell carcinoma; KIRP: kidney renal papillary cell carcinoma; LAML: acute myeloid leukemia; LGG: brain lower-grade glioma; LIHC: liver hepatocellular carcinoma; LUAD: lung adenocarcinoma; LUSC: lung squamous cell carcinoma; MESO: mesothelioma; OV: ovarian serous cystadenocarcinoma; PAAD: pancreatic adenocarcinoma; PCPG: pheochromocytoma and paraganglioma; PRAD: prostate adenocarcinoma; READ: rectum adenocarcinoma; SARC: sarcoma; SKCM: skin cutaneous melanoma; STAD: stomach adenocarcinoma; TGCT: testicular germ cell tumor; THCA: thyroid carcinoma; THYM: thymoma; UCEC: uterine corpus endometrial carcinoma; UCS: uterine carcinosarcoma; UVM: uveal melanoma.

Pan-cancer data downloaded from TCGA database were used to evaluate FEN1 mRNA expression levels. A comparison between diseases and healthy tissues showed that expression of FEN1 mRNAs was universally upregulated in most tumor tissues (Figure 1(a)), including HCC (*P* < 0.001) [12]. Similarly, analysis of protein expression in the pan-cancer TMA showed a high FEN1 expression in HCC compared to adjacent normal tissues (Figures 1(b) and 1(c)). These results indicated that FEN1 protein is upregulated in many tumors, including HCC.

### 3.2. FEN1 mRNA Levels Are Significantly Overexpressed in TCGA and GEO HCC Datasets

**Figure 2:**
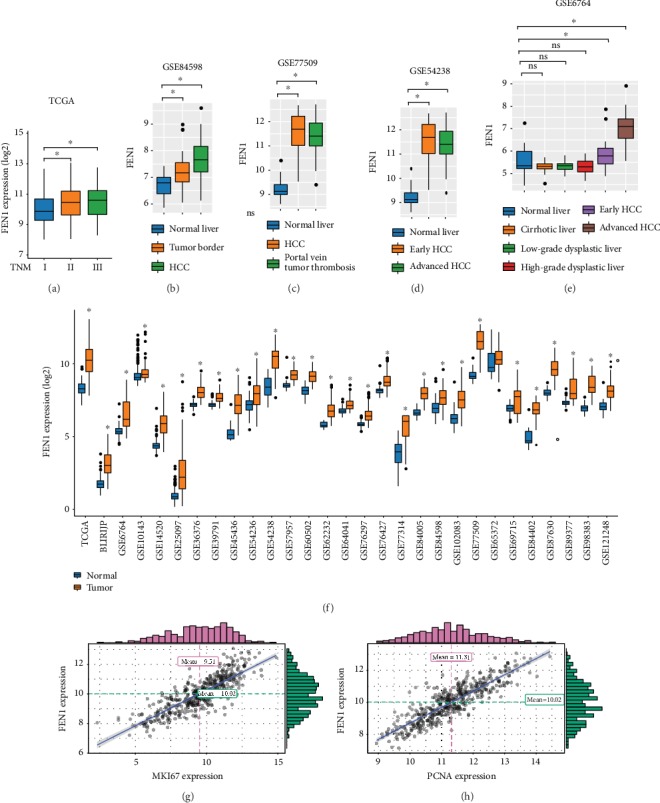
FEN1 mRNA is significantly upregulated in HCC tissues. (a-e) The correlation of FEN1 expression with the TNM stage and histologic grade in TCGA and four GEO databases. (f) FEN1 mRNA expression in HCC tissues and paired nontumor tissues of TCGA and GEO databases. (g-h) The correlation of FEN1 expression with MKI67 and PCNA. FEN1: flap endonuclease 1; HCC: hepatocellular carcinoma; TMA: tissue microarrays; GEO: Gene Expression Omnibus; TCGA: The Cancer Genome Atlas; GSEA: gene set enrichment analysis; PCNA: proliferating cell nuclear antigen.

Based on the findings of FEN1 expression in pan-cancer, we further evaluated FEN1 mRNA expression in HCC and peritumoral tissues using TCGA and GEO HCC database. We found that FEN1 mRNA expression is associated with the TNM stage and histologic grade of HCC in TCGA and four GEO databases (Figure 2(a)). To further ascertain the profiles of FEN1 mRNA expression in HCC, we analyzed TCGA database and sixteen independent microarray datasets of HCC from the GEO database and observed similar results (Figure 2(b)). Moreover, there was a positive correlation between the levels of FEN1, MKI67 (*R* = 0.84, *P* < 0.001), and PCNA (*R* = 0.87, *P* < 0.001), indicating that FEN1 overexpression induced proliferation in HCC cells (Figure 2(c)). These results corroborated our earlier observations that expression of FEN1 mRNA is significantly elevated in HCC tissues.

### 3.3. FEN1 Proteins Are Significantly Overexpressed in the HCC TMA Cohort

**Figure 3:**
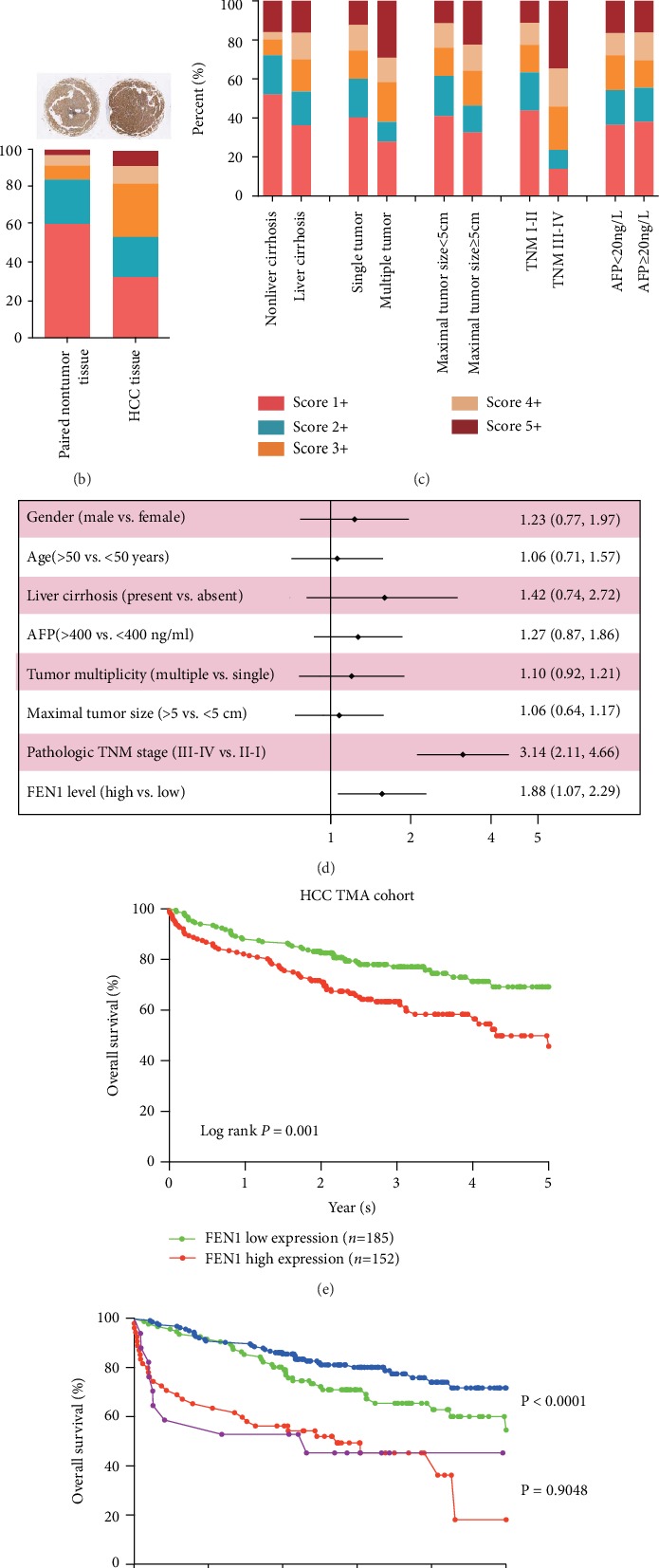
FEN1 protein is significantly upregulated in our HCC TMA cohort. (a) Representative FEN1 and immunohistochemical staining patterns with different staining scores in HCC tissues. (b) Representative FEN1 histologic scoring in HCC tissues and paired nontumor samples. (c) Distribution of FEN1 immunohistochemical staining scores in HCC tissues according to liver cirrhosis, tumor multiplicity, maximal tumor size, venous invasion, pathologic TNM stage, and AFP level. (d) Forest plot depicting correlations between the indicated clinical criteria and the FEN1 staining scores. (e) The overall survival analysis of FEN1 expression in the HCC TMA cohort. (f) The overall survival analysis between different degrees of FEN1 and different degrees of the TNM stage. FEN1: flap endonuclease 1; HCC: hepatocellular carcinoma; TMA: tissue microarrays; HE: hematoxylin-eosin staining; TNM: tumor, lymph node, metastasis.

Considering the difference between mRNA transcription and protein expression, we evaluated protein changes of FEN1 in HCC using TMA containing 396 pairs of HCC samples. We detected FEN1 proteins using IHC analysis, and using staining intensity and percentage of positive cells in tissue sections, we categorized FEN1 staining patterns into five scores (Figure 3(a)). FEN1 protein expression was higher in HCC tissues relative to other tissues (Figure 3(b)). Analysis of patients' score distribution across different clinical features indicated higher FEN1 expression and a positive relationship with the advanced TNM stage (Figure 3(c)). Importantly, results from the multivariate analysis showed that the TNM stage and high FEN1 expression were independent prognostic indicators for overall survival in HCC patients studied (Figure 3(d) and Tables 1 and 2). Meanwhile, Kaplan-Meier analysis indicated that the overall survival time was shorter in HCC patients with higher FEN1 expression (Figures 3(e) and 3(f)). Taken together, these results confirmed that FEN1 protein levels were remarkably upregulated in HCC and were closely associated with prognosis of HCC patients.

### 3.4. Effect of FEN1 Upregulation on Disease Outcome in TCGA HCC Cohort

We further correlated FEN1 expression and corresponding patients' clinical-pathologic features and analyzed the relationship across TCGA HCC cohort. These relationships are summarized in detail in Table 3. Overall, we found a positive correlation between high expression of FEN1 in tumor tissues and the late TNM stage. Univariate analyses showed that FEN1 expression and the TNM stage had a significant negative correlation with OS of HCC patients. FEN1 levels were not correlated with gender, age, race, or tumor grade (Table 4). Notably, consistent with the results from our HCC patients, a multivariate analysis showed that FEN1 was an independent prognostic predictor for RFS (*P* = 0.025).

**Figure 4:**
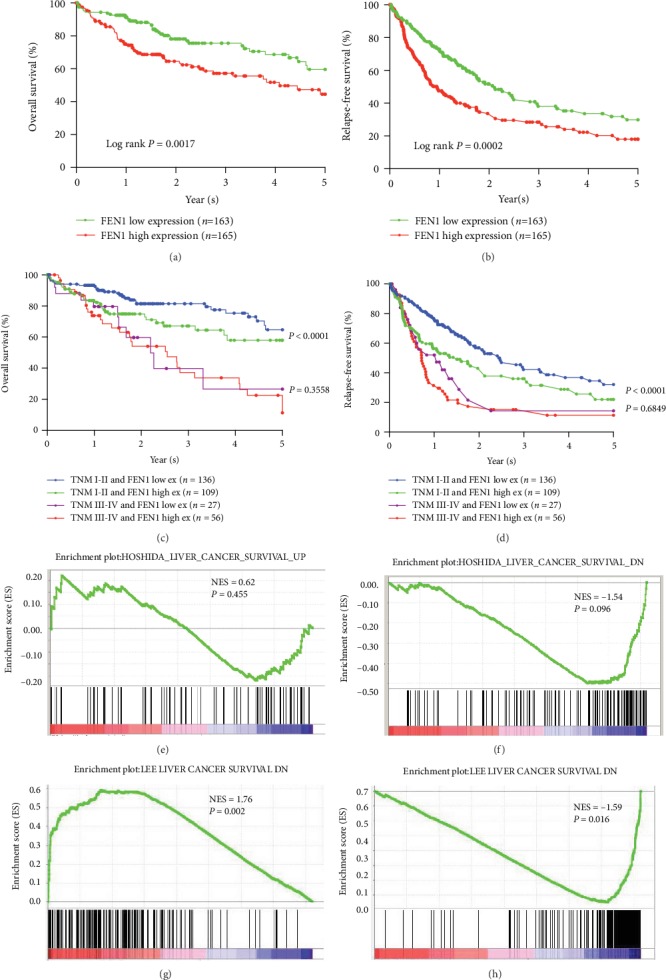
Analysis of the relationships between FEN1 expression and prognosis in HCC TCGA samples. (a) Kaplan-Meier overall survival analysis between different degrees of FEN1 expression. (b) Kaplan-Meier relapse-free survival analysis between different degrees of FEN1 expression. (c, d) Kaplan-Meier overall survival analysis between different degrees of FEN1 and different degrees of the TNM stage. (e–h) The gene set enrichment analysis from TCGA HCC dataset revealed a high expression of FEN1 correlated with gene signatures of poor survival. FEN1: flap endonuclease 1; HCC: hepatocellular carcinoma; GEO: Gene Expression Omnibus; TCGA: The Cancer Genome Atlas; NES: not otherwise specified.

To further evaluate the prognostic value of FEN1, the relationship between FEN1 expression and corresponding clinical follow-up information was analyzed in TCGA HCC cohort. Results showed that patients who had a high level of FEN1 also had lower rates of OS and RFS compared to those who had low FEN1 levels (Figures 4(a) and 4(b)). Moreover, HCC patients at the high stage (TNM III-IV) and with high expression of FEN1 were prone to low OS (Figure 4(c)) and RFS (Figure 4(d)) rates. Results from GSEA for TCGA HCC dataset showed that the high FEN1 expression was associated with poor survival of gene signatures (Figures 4(e)–4(h)). These results further confirmed that FEN1 was overexpressed in HCC and positively correlated with poor prognosis in HCC patients.

### 3.5. FEN1 as a Panel of Biomarker in the Diagnosis of HCC

**Figure 5:**
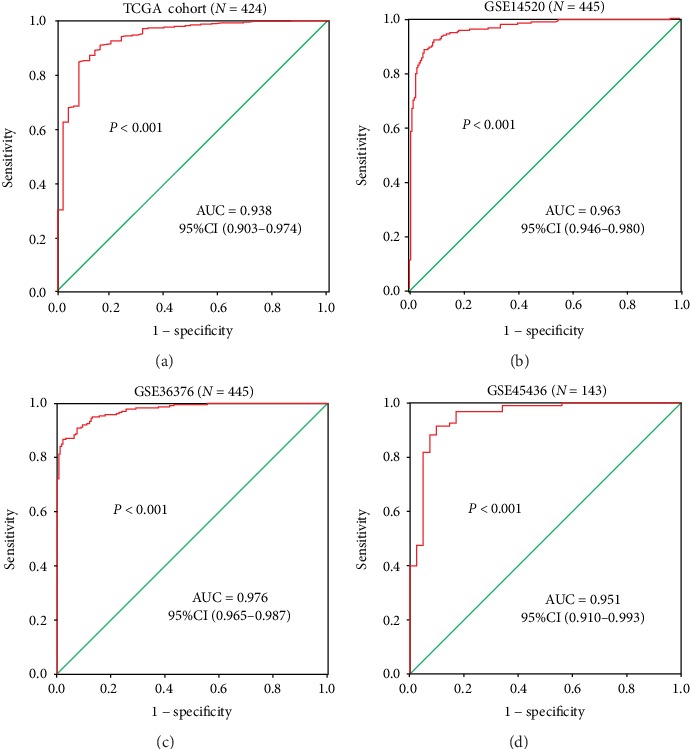
ROC analysis of FEN1 in HCC. (a) AUC of FEN1 in HCC patients from TCGA databases. (b–d) AUC of FEN1 in HCC patients from GEO14520, GEO36376, and GEO45436. AUC: area under the curve; FEN1: flap endonuclease 1; HCC: hepatocellular carcinoma; GEO: Gene Expression Omnibus; TCGA: The Cancer Genome Atlas; ROC: receiver operating characteristic; CI: confidence interval.

To explore the clinical significance of FEN1 in distinguishing hepatocellular carcinoma tissues from normal ones in the liver, we analyzed the receiver operating characteristic (ROC) in four independent HCC cohorts. The ROC curve with TCGA HCC cohort (Figure 5) showed that the area under the curve (AUC) was 0.938 (95% CI: 0.903-0.974, *P* < 0.001) with sensitivity of 0.847 and specificity of 0.922 (Figure 5(a)). Validation of FEN1's diagnostic value in other three HCC cohorts showed that the AUC levels were 0.963 (*P* < 0.001), 0.976 (*P* < 0.001), and 0.951 (*P* < 0.001) in GSE14520 (Figure 5(b)), GSE36376 (Figure 5(c)), and GSE45436 (Figure 5(d)) databases, respectively. This was consistent with TCGA results and indicated that FEN1 can be a biomarker for the diagnosis of HCC patients.

### 3.6. The Potential Mechanism of FEN1 Action in HCC

**Figure 6:**
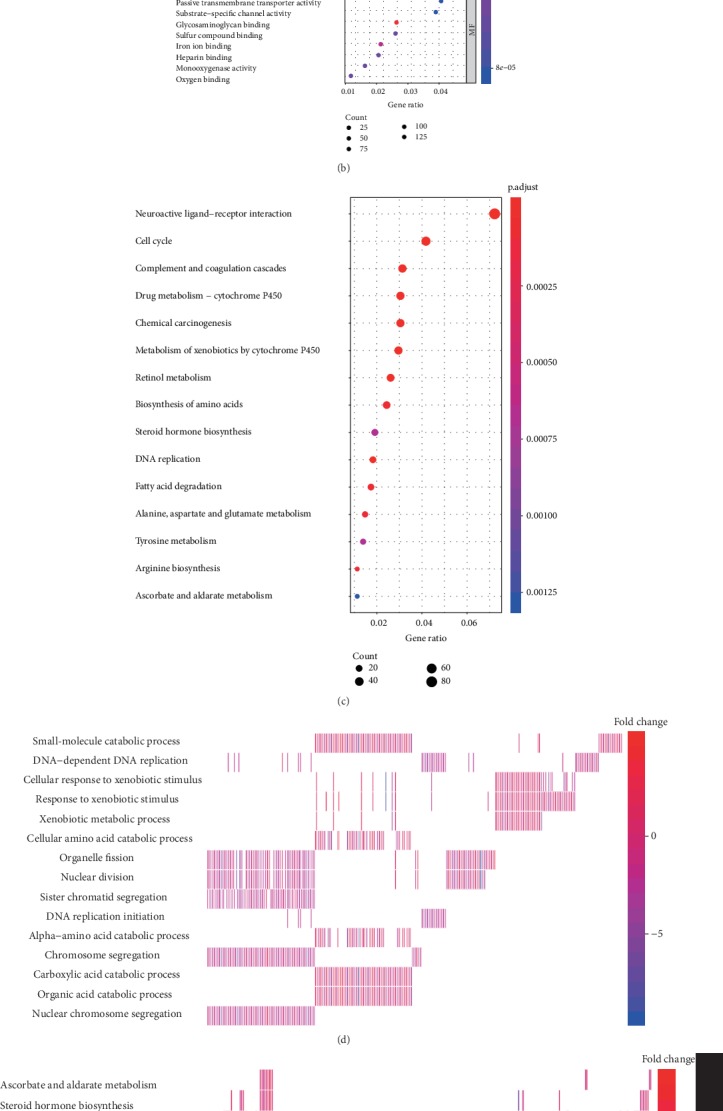
Functional and pathway enrichment analysis. (a) The heat map of the differential expression analysis between FEN1 high- and low-expression groups. (b) GO (Gene Ontology) enrichment analysis of differentially expressed FEN1 genes. (c) KEGG enrichment analysis of differentially expressed FEN1 genes. (d, e) Heat map of enrichment analysis of differentially expressed FEN1 genes. (f) Correlation analysis between FEN1 and cell cycle pathway-related genes. (g) Correlation analysis between FEN1 and DNA replication pathway-related genes. FEN1: flap endonuclease 1; HCC: hepatocellular carcinoma; GO: Gene Ontology; NES: not otherwise specified.

The possible mechanisms of FEN1 in HCC were evaluated by functional and pathway enrichment analyses. The heat maps of the differential expression analysis (DEGs) are shown in Figure 6(a). According to cluster profile GO and KEGG analyses, we found that the cell cycle and DNA replication pathways are significantly enriched in samples with high FEN1 expression (Figures 6(b)–6(e)). We hypothesized that FEN1 might be contributing to HCC development by regulating the cell cycle and DNA replication. In this regard, we performed a correlation analysis between FEN1 and the genes related to the cell cycle and DNA replication pathways. The result showed that there was a positive correlation between the levels of FEN1 expression and cell cycle pathway-related genes, including CDK1 (*R* = 0.85), CDK2 (*R* = 0.69), CDK3 (*R* = 0.45), CDK4 (*R* = 0.7), CDK5 (*R* = 0.56), and CDK7 (*R* = 0.56) (Figure 6(f)). Moreover, FEN1 expression had a positive correlation with DNA replication pathway-related genes (Figure 6(g)), including DNA2 (*R* = 0.7), MCM2 (*R* = 0.9), MCM3 (*R* = 0.87), MCM4 (*R* = 0.83), MCM5 (*R* = 0.88), MCM6 (*R* = 0.88), and MCM7 (*R* = 0.87). These results suggested that the potential mechanism of action of the FEN1 gene was related to its role in the cell cycle and DNA replication in HCC.

### 3.7. Knockdown of FEN1 Inhibits HCC Progression *In Vitro*

**Figure 7:**
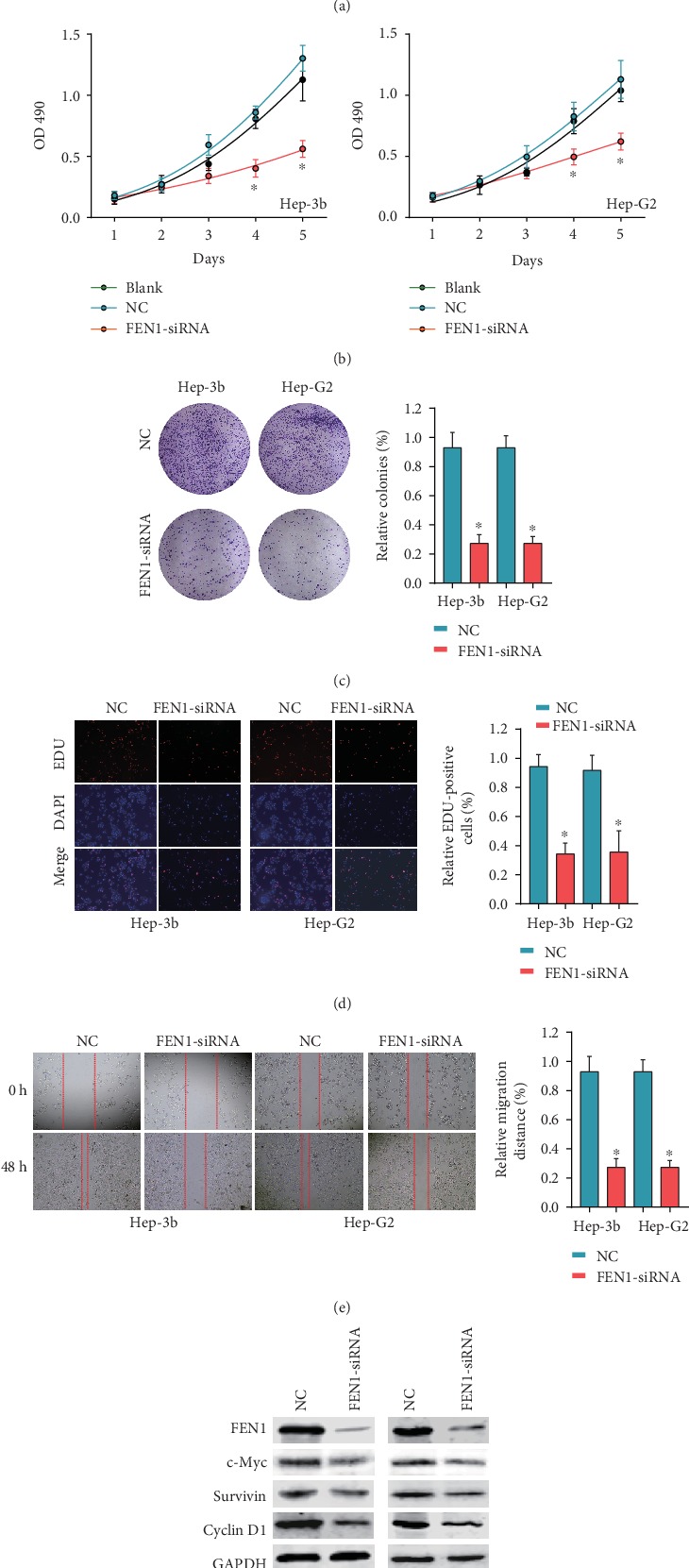
FEN1 promotes cell proliferation and migration in vitro. (a) The protein levels of FEN1 in Hep-3b and Hep-G2 after knockdown by si-FEN1 were detected by western blot. (b) Knockdown of FEN1 in Hep-3b and Hep-G2 significantly decreased cell proliferation compared with si-NC cells, performed by the CCK-8 assay. (c) Colony numbers of Hep-3b and Hep-G2 cells transfected with si-FEN1 were significantly lower than those transfected with si-NC, measured using the colony formation assay. (d) Suppression of FEN1 attenuated the proliferation of Hep-3b and Hep-G2 cells, measured by the EDU assay (magnification, ×100). Scale bar, 100 *μ*m. (e) FEN1 suppression impaired HCC cell migration, as measured by the wound healing assay. ^∗^*P* < 0.05, ^∗∗^*P* < 0.01, and ^∗∗∗^*P* < 0.001. Error bars indicate mean ± SD. (f) Protein levels of FEN1, c-Myc, survivin, and cyclin D1 after knockdown by si-FEN1. FEN1: flap endonuclease 1; HCC: hepatocellular carcinoma.

We further transfected Hep-3b and Hep-G2 cells with FEN1-siRNAs and si-NC and used the western blot assay to confirm the efficiency of transfection (Figure 7(a)). Consequently, we found that knockdown of FEN1 significantly inhibited cell proliferation (Figure 7(b)). Analysis of colony formation showed a marked decrease in the number of colonies after FEN1 depletion (Figure 7(c)). The EDU assay demonstrated that FEN1 suppressed proliferation of HCC cells (Figure 7(d)). In addition, the wound healing assays revealed suppressed migration in HCC cells transfected with si-FEN1 (Figure 7(e)). Overall, knockdown of FEN1 reduced the expression of c-Myc, survivin, and cyclin D1 (Figure 7(f)) suggesting that FEN1 is an oncogene of HCC that regulates the cell cycle and DNA replication.

## 4. Discussion

Maintenance of genomic integrity through DNA repair is an essential component of healthy cell homeostasis and is indispensable in cell growth, differentiation, and apoptosis [13]. Studies have shown that FEN1 has a dual function in DNA replication and repair, and its expression levels and functional disorder can induce genomic instability, leading to cancer development [10, 14, 15]. However, expression profiles and functional significance of the FEN1 gene have not been studied in HCC. In this study, we found a high expression of FEN1 in HCC cells and this was associated with poor prognosis in patients diagnosed with the condition. In addition, the expression of FEN1 resulted in a high AUC value which effectively distinguished HCC tumors from normal liver tissues. FEN1 knockdown resulted in significantly suppressed proliferation and migration of HCC cells *in vitro*. These results suggest that FEN1 was highly expressed in HCC and might serve as a potential biomarker for prognosis and diagnosis of HCC patients besides being a therapeutic target for improving HCC prognosis.

Numerous studies have described the role played by FEN1 in tumor formation and proliferation. This gene has been reported to be upregulated in many human malignancies including non-small-cell lung cancer [16], breast cancer [17], prostate cancer [18], gastric cancer [19], colorectal cancer [20], and cervical cancer [21]. In the current study, we detected a higher expression of FEN1 in hepatocellular carcinoma tissues relative to adjacent nontumor tissues. This was confirmed not only via TCGA and GEO HCC databases but also by analysis of HCC TMA.

Previous studies have reported that overexpression of FEN1 is positively correlated with poor prognosis of non-small-cell lung cancer [16] and breast cancer [17], as well as lung adenocarcinoma [22]. In addition, the gene has been proposed as a prognostic biomarker for cancer. For instance, Abdel-Fatah et al. [23] described FEN1 as a promising biomarker in breast and ovarian epithelial cancers confirming its potential as a prognostic biomarker for malignant tumors. In the present study, we found that HCC patients, who showed high expression of FEN1, had shorter overall survival time and shorter relapse-free survival time, in line with previous studies. Meanwhile, analysis of the ROC curve indicated a high diagnostic efficacy of FEN1 as it was capable of differentiating the HCC tissue from normal liver tissues. Overall, these findings indicate that overexpression of FEN1 might serve as a promising prognostic and diagnostic biomarker for HCC patients. FEN1 inhibitors should, therefore, be considered potential novel drugs for HCC [24].

We also predicted the potential mechanism of FEN1 action in HCC using functional and pathway enrichment analyses with the results showing that high FEN1 expression was related to HCC cell cycle and DNA replication pathways. Specifically, FEN1 expression was positively correlated with cell cycle and DNA replication pathway-related genes, as well as the malignant phenotype in HCC. *In vitro* experiments proved that silencing FEN1 inhibited cell proliferation and migration by regulating expressions of c-Myc, survivin, and cyclin D1. These findings are consistent with previously reported roles of FEN1 in both DNA replication and repair [5]. Collectively, our results indicated that the potential mechanism of FEN1 action in HCC might be regulating the cell cycle and DNA replication pathway.

## 5. Conclusion

In summary, we found that FEN1 was overexpressed in HCC cells and positively associated with poor prognosis of HCC patients. In addition, FEN1 expression shows high capacity to differentiate HCC tissue from noncancer liver tissues. Taken together, these findings indicate that FEN1 is a potential prognostic and diagnostic biomarker for HCC patients.

## Figures and Tables

**Table 1:** The relationship of FEN1 expression in HCC with clinicopathological characteristics.

Characteristics		FEN1		
		Low	High	*P* value
Sex	Female	136 (51.9%)	126 (48.1%)	0.715
	Male	37 (49.3%)	38 (50.7%)	

Age	<50	65 (52%)	60 (48%)	0.851
	≥50	108 (50.9%)	104 (49.1%)	

Liver cirrhosis	Present	160 (51.3%)	152 (48.7%)	0.945
	Absent	13 (52%)	12 (48%)	

AFP	<20	82 (50%)	82 (50%)	0.633
	≥20	91 (52.6%)	82 (47.4%)	

Tumor multiplicity	Multiple	43 (54.4%)	36 (45.6%)	0.529
	Single	130 (50.4%)	128 (49.6%)	

Pathologic TNM stage	I/II	168 (63.4%)	97 (36.6%)	<0.001
	III/IV	17 (23.61%)	55 (76.39%)	

**Table 2:** Univariate and multivariate Cox regression analyses of risk factors for overall survival rate (*n* = 337).

			Univariate analysis			Multivariate analysis		
		OS (%)	HR	95% CI	*P* value	HR	95% CI	*P* value
Age	<50 (*n* = 125)	87 (69.6%)	1.060	0.714–1.573	0.773			
	≥50 (*n* = 212)	142 (67.0%)						

Gender	Female (*n* = 75)	53 (70.7%)	1.231	0.770-1.967	0.385			
	Male (*n* = 262)	176 (67.2%)						

Liver cirrhosis	Absent (*n* = 25)	15 (60%)	1.420	0.740-2.722	0.291			
	Present (*n* = 312)	214 (68.6%)						

AFP	<20 (*n* = 164)	118 (72.0%)	1.269	0.866-1.860	0.222			
	≥20 (*n* = 173)	111 (64.2%)						

Tumor multiplicity	Single (*n* = 258)	174 (67.4%)	1.095	0.916-1.209	0.696			
	Multiple (*n* = 79)	55 (69.6%)						

Maximal tumor size	<5 cm (*n* = 190)	127 (66.8%)	1.063	0.638-1.174	0.756			
	≥5 cm (*n* = 147)	102 (69.4%)						

Pathologic TNM stage	I/II (*n* = 265)	196 (74.0%)	3.136	2.112-4.658	<0.001	3.161	2.129-4.695	<0.001
	III/IV (*n* = 72)	33 (45.8%)						

FEN1 level	Low (*n* = 173)	126 (72.8%)	1.881	1.066-2.285	0.022	1.585	1.083-2.320	0.018
	High (*n* = 164)	103 (62.8%)						

**Table 3:** Relationship between clinicopathological variables and FEN1 expression level in TCGA database.

Characteristics		FEN1		
		Low	High	*P* value
Gender	Male	123 (54.9%)	101 (45.1%)	0.006
	Female	40 (38.5%)	64 (61.5%)	

Age	<Median	76 (47.2%)	85 (52.8%)	0.376
	≥Median	87 (52.1%)	80 (47.9%)	

Race	White	90 (56.6%)	69 (43.4%)	0.015
	Others	73 (43.2%)	96 (56.8%)	

TNM stage	Stage I/II	136 (55.5%)	109 (44.5%)	<0.001
	Stage III/IV	27 (32.5%)	56 (67.5%)	

Histologic grade	Grade I/II	117 (58.2%)	84 (41.8%)	<0.001
	Grade III/IV	46 (36.2%)	81 (63.8%)	

AFP	Low	99 (63.1%)	58 (36.9%)	<0.001
	High	64 (37.4%)	107 (62.6%)	

**Table 4:** Univariate and multivariate analysis of FEN1 associated with OS and RFS in TCGA HCC cohort.

			Univariate analysis			Multivariate analysis		
		OS (%)	HR	95% CI	*P* value	HR	95% CI	*P* value
Univariate and multivariate analyses of overall survival in HCC patients (*n* = 328)								
Age	<Medium (*n* = 161)	116 (72.1%)	0.822	0.580–1.165	0.270			
	≥Medium (*n* = 167)	112(67.1%)						
Gender	Female (*n* = 105)	67 (63.8%)	1.117	0.936-1.334	0.220			
	Male (*n* = 223)	161(72.2%)						
Race	Others (*n* = 169)	122 (72.2%)	1.454	0.800-2.642	0.220			
	White (*n* = 159)	106 (66.7%)						
TNM stage	Stage I/II (*n* = 245)	187 (76.3%)	2.483	1.713-3.600	<0.001	2.271	1.544-3.339	<0.001
	Stage III/IV (*n* = 83)	41 (49.4%)						
AFP expression	Low (*n* = 157)	115 (73.3%)	0.859	0.608-1.215	0.391			
	High (*n* = 171)	113 (66.1%)						
FEN1 expression	Low (*n* = 163)	124 (76.1%)	1.606	1.133-2.277	0.008	1.378	0.935-2.029	0.105
	High (*n* = 165)	104 (63.0%)						

Univariate and multivariate analyses of relapse-free survival in HCC patients (*n* = 328)								
Age	<Medium (*n* = 161)	116 (72.1%)	0.965	0.845-1.102	0.601			
	≥Medium (*n* = 167)	112 (67.1%)						
Gender	Female (*n* = 105)	67 (63.8%)	1.053	0.916-1.209	0.468			
	Male (*n* = 223)	161(72.2%)						
Race	Others (*n* = 169)	122 (72.2%)	0.865	0.638-1.174	0.352			
	White (*n* = 159)	106 (66.7%)						
TNM stage	Stage I/II (*n* = 245)	187 (76.3%)	2.066	1.538-2.775	<0.001	1.883	1.388-2.556	<0.001
	Stage III/IV (*n* = 83)	41 (49.4%)						
AFP expression	Low (*n* = 157)	115 (73.3%)	0.846	0.650-1.102	0.215			
	High (*n* = 171)	113 (66.1%)						
FEN1 expression	Low (*n* = 163)	124 (76.1%)	1.593	1.221-2.078	0.001	1.387	1.043-1.845	0.025
	High (*n* = 165)	104 (63.0%)						

## Data Availability

The data used to support the findings of this study are downloaded from TCGA database and GEO database. As mentioned in Materials and Methods, sixteen HCC mRNA expression datasets (GSE6474, GSE10143, GSE39791, GSE45436, GSE14520, GSE36376, GSE54236, GSE60502, GSE76297, GSE76427, GSE62232, GSE64041, GSE77314, GSE84005, GSE84598, and GSE102083) were obtained from the Gene Expression Omnibus (GEO) database (http://www.ncbi.nlm.nih.gov/geo/). TCGA-LIHC and corresponding clinical data used in this study were downloaded from The Cancer Genome Atlas (TCGA) data portal (https://gdc-portal.nci.nih.gov/).
